# Nutritional status of Zombi pea (*Vigna vexillata*) as influenced by plant density and deblossoming

**DOI:** 10.1038/s41598-024-52736-7

**Published:** 2024-02-07

**Authors:** Srija Priyadarsini, Alok Nandi, Maniyam Nedunchezhiyan, Pushpajeet Choudhari, Saurabh Singh, Ajoy Pattnaik

**Affiliations:** 1 Department of Vegetable Science, Institute of Agricultural Sciences, Siksha ‘O’ Anusandhan (Deemed to be University), Bhubaneswar, Odisha 751029 India; 2 Central Tuber Crop Research Institute (CTCRI), Regional Centre, Bhubaneswar, Odisha 751019 India; 3 International Crops Research Institute for the Semi-Arid Tropics, Patancheru, Hyderabad, Telangana 502324 India; 4 Department of Vegetable Science, Rani Lakshmi Bai Central Agricultural University, U.P., Jhansi, 284003 India

**Keywords:** Agroecology, Biodiversity, Plant sciences, Climate sciences

## Abstract

Feeding billions, a healthy and nutritious diet in the era of climate change is a major challenge before plant breeders, geneticists and agronomist. In this context, the continuous search for adaptive and nutritious crops could be a better alternative to combat the problems of hunger and malnutrition. The zombi pea, a nutritious and underutilized leguminous vegetable, is one of such better alternatives to feed billions a nutritious food besides being a potential gene source for breeding abiotic stress resistant varieties. To evaluate its potential as a wonder crop in the tropical and subtropical regions of India, the nutritional status of tubers, pods and pericarp were investigated under different treatments of plant spacings and deblossoming. The experiment was conducted in split plot design with three replications and eight treatments during 2021–2022 in the coastal regions of India. The nutrient profiling in tubers and pericarp of pods in zombi pea revealed higher accumulation of nutrients viz. potassium (K), magnesium (Mg), iron (Fe), manganese (Mn) and zinc (Zn) with blossom retention. The zombi pea tubers reflected significantly high protein accumulation with the increase in plant spacing. The results pertaining to nutrient profiling in the pods of zombi pea indicated that the plant spacing has no significant effect on the accumulation of majority of nutrients under study. The above-mentioned findings are conspicuously novel and valuable. The present study would pave the way for understanding nutritional importance and breeding potential of this orphan crop. The blossom retention renders higher nutrient accumulation in tubers, pods and pericarp of zombi pea. Deblossoming has no significant influence on nutritional profile of this wonder crop but, wider spacing is effective in producing tubers with high protein content.

## Introduction

Micronutrient deficiencies such as zinc (Zn), iron (Fe), selenium (Se), magnesium (Mg), copper (Cu), and iodine (I), are a widespread and growing problem in both crop plants and human populations worldwide^1^. Insufficient amounts and low bioavailability of micronutrients in plant-based diets represent a major reason for the high prevalence of micronutrient deficiencies in humans. About one-third of the human population is globally affected by the micronutrient deficiencies (also known as ‘hidden hunger’) which are attributed to low dietary intake, mainly in developing regions where populations rely on plant-based diets from micronutrient poor cereal crops^2,3^. Low dietary intake of micronutrients is also widely reported in European populations, especially among elderly people and children, with significant adverse impacts on health. In addition, the high alcohol consumption that is common in Europe significantly interferes with the intestinal absorption of micronutrients and contributes to Zn, Fe, and Se deficiencies in human populations^4,5^. Various approaches can be adopted for combating the micronutrient deficiencies and delivering a micronutrient enriched diet at door step: (i) biofortification of major and minor crops through agronomic and breeding strategies (ii) screening of orphan or indigenous crop species or macroalgae for micronutrient content and availability, and their introduction into the daily human diet^6,7^ (iii) The crop plant species with abundant micronutrient content, like Zn-hyperaccumulating species, can be cultivated and used as an additive in other foods. Zombi pea is one of the potential underutilized leguminous vegetable crops and which can be harnessed for combating problems of malnutrition and poverty^8^.

**Figure 1:**
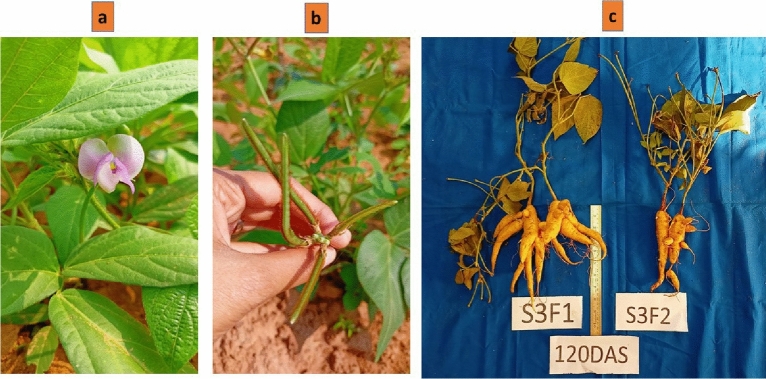
Zombi pea plant morphology. (**a**) flower of zombi pea (**b**) pods of zombi pea (**c**) plant root tubers.

Zombi pea (*Vigna vexillata* (L.), 2n = 2x = 22), a pan-tropical herbaceous legume crop, is widely distributed in Southern and Eastern Africa, the Indian subcontinent, South-East Asia, Indonesia, Papua New Guinea, and Australia^9,10^. *Vigna vexillata* has been recorded in two domesticated varieties: the seed type and the tuber (storage root) type. The tuber type is believed to have originated in the Indonesian region that extends to India, whereas the seed type is supposed to have been domesticated in Sudan (Africa)^11–13^. This legume crop, also known as tuber cowpea, wild cowpea, etc., is a potentially useful underutilized crop that produces edible green pods and root tubers on the same plant^14^. With trifoliate leaves and axillary inflorescences, *Vigna vexillata* is a prostrate, spreading, pubescent, annual legume (Fig. 1). It is a wild species used for its storage roots, protein-rich seed and also as a forage and erosion control plant^15,16^. The edible tubers of the species have a protein content of about 15%, which is approximately three times more than that of potato and yam and six times higher than that of cassava^13^. Throughout the flowering stage, the herbage of *Vigna vexillata* var. *vexillata* is quite nutrient-rich with a crude protein level of 20.3%^17^. Negi and Gaur^18^ revealed that 14.5% protein was present in the dry roots of *Vigna vexillata*. The researchers have reported that the *Vigna vexillata* (L.) A. Rich var *vexillata* contains good amounts of Fe (438.5 µg/g), Zn (76 µg/g), Cu (126.5 µg/g) and Mn (142.5 µg/g), making it a good source of micronutrients^19^.The expanded forms of abbreviations and unique symbols are presented in supplementary Table S1.

Plant population density is a major determinant of crop yield per unit of land area and is a dependent variable of plant spacing. Narrow row spacing reduces weed germination and growth and gives the crop a competitive advantage over weeds due to rapid canopy closure^20^. The rapid canopy development at narrow row spacing might be attributed to more light interception per unit leaf area index, thereby increasing photosynthetic rates of the leaves and hence, better growth and development occurs^21^. The primary organs of plants are their flowers, which have a significant impact on the distribution of dry matter and sugar, their accumulation, fruit quality, and changes in endogenous hormones^22–24^. Removal of the flowers may affect the distribution of carbohydrates to the tubers, increase tuber production and serve as a paradigm for high yield agriculture. The increase in tuber biomass and potential utility as bioenergy were both influenced by the enhancements of C and N contents^25^.

Among the various domesticated species of *Vigna*, tuber cowpea is one of the least researched crops for its genetic resources, and production technologies, especially in the Indian subcontinent^26^. This crop is gaining popularity gradually, and researchers have identified the QTLs associated with domestication syndrome^13,27^. QTLs, for bruchid resistance has been identified in zombi pea using ‘TVNu 240’, a resistant line^28^. Elaborative research must be conducted to standardize production technologies of the crop. Keeping in mind the high nutritional and economical potential of tuber cowpea, the present investigation was conducted to analyse that how spacing and deblossoming affect micronutrient availability in this orphan crop, with the intent of not only increasing output and correcting micronutrient deficiencies in humans, but also boosting agricultural sustainability. The study was planned to understood the influence of variable spacing and deblossoming in nutrient status of pods and tubers of zombi pea. The influence of zombi pea under different treatments was investigated on various soil parameters as well. This study is first of its kind in India regarding analysis of nutritional profiling of this underutilized neglected crop.

## Materials and methods

### Experimental location and plant material

The field experiment was conducted during the 2021–2022 at the Regional Centre of ICAR-Central Tuber Crops Research Institute (CTCRI) (20.1450◦ N and 85.4706◦ E), Dumduma, Bhubaneswar, Odisha, India. The estimation of nutritional traits was carried out at the Charles Renard Analytical Laboratory (CRAL), ICRISAT. Growth parameter analysis was carried out at the Siksha ‘O’ Anusandhan (deemed to be university), Bhubaneswar, Odisha. The plant material comprised of Zombi pea line ‘IC 259504’ which is indeterminate in growth habit and was provided by ICAR-CTCRI, Regional Centre, Bhubaneswar and is registered at ICAR-National Bureau of Plant Genetic Resources (NBPGR), New Delhi. The material can be accessed from ICAR-NBPGR and is a cultivated form of Zombi pea. The field work was done at ICAR-CTCRI, Regional Centre only.

### Experimental design and nutritional traits under study

The experiment was conducted in the Split plot design with 8 treatments and 3 replications. There were 4 levels of spacings (S_1_-45 cm × 15 cm, S_2_- 45 cm × 30 cm, S_3_- 60 cm × 15 cm and S_4_- 60 cm × 30 cm) in the main plot and 2 levels of deblossoming (F_1_-flower removal and F_2_-flower retention) treatments in the sub plot (Table 1) The plan of layout is depicted in Table 2. All the flowers were removed during deblossoming (F_1_) manually 60 days after sowing till peak flowering period. The sulfuric acid-selenium digestion approach as suggested by Sahrawat et al.^29^ with minor modifications was employed for the analysis of protein, micro nutrients (Fe, Mn, Zn, Cu), macro (N, P, K) and secondary nutrients (Ca, Mg) in the tuber, pod and pericarp of zombi pea at ICRISAT Hyderabad, India. For the estimation of different nutrient levels in single digestion, sulfuric acid-selenium digestion mixture was utilized. Starch and sugar analyses were done as per the procedure presented by Stickland and Wilson^30^ and Weng et al.^31^ with minor modifications.

**Table 1:** Treatment details.

Sr. no.	Treatment	Symbol used
a.	Main plot (Spacing)	
	1. 45 × 15 cm	1. S_1_
	2. 45 × 30 cm	2. S_2_
	3. 60 × 15 cm	3. S_3_
	4. 60 × 30 cm	4. S_4_
b.	Sub plot (Deblossoming)	
	1. Inflorescence removal	1. F_1_
	2. Inflorescence retention	2. F_2_
c.	Interactions	1. S_1_F_1_
		2. S_1_F_2_
		3. S_2_F_1_
		4. S_2_F_2_
		5. S_3_F_1_
		6. S_3_F_2_
		7. S_4_F_1_
		8. S_4_F_2_

**Table 2:** Plan of layout.

R1	S_4_F_2_	S_3_F_2_	S_2_F_1_	S_1_F_1_
	S_4_F_1_	S_3_F_1_	S_2_F_2_	S_1_F_2_
R2	S_1_F_1_	S_2_F_2_	S_3_F_2_	S_4_F_1_
	S_1_F_2_	S_2_F_1_	S_3_F_1_	S_4_F_2_
R3	S_2_F_2_	S_1_F_2_	S_4_F_1_	S_3_F_2_
	S_2_F_1_	S_1_F_1_	S_4_F_2_	S_3_F_1_

### Sampling technique and nutrient analysis

Five plants from each plot were taken randomly excluding border plants and were tagged as sample plants for recording observations. About 0.5 g finely ground plant samples were taken in 250 ml digestion tube. Then, 14 ml of concentrated sulfuric acid comprising 0.5% selenium powder was immersed into each of the digestion tube to soak the grounded plant samples. For the preparation of Sulfuric acid-Se mixture, Se powder was dissolved in concentrated sulfuric acid by heating on a hot plate with intermittent stirring with a glass rod. The mixture was allowed to cool and final volume was made up to one litre. The digestion tubes were shifted to preheated block digester and the full digestion takes about 2.5 h which is indicated by colourless and clear plant digests after digestion. In case of Ca estimation, the dilution of plant digests with 30–40 ml of distilled water by keeping overnight is practiced. This is done to avoid the insoluble calcium sulfate. All the plant nutrient analysis were performed in triplicate as results reported as average values were analysed^29^ The digests volume was made up to 250 ml with distilled water. Then aliquots of digests were used for respective estimation of nutrients like distillation with NaOH for N determination, phosphovanadomolybdate colorimetric method for the estimation of P and atomic absorption spectrophotometer for the determination of micronutrients like K, Ca, Mg etc^29^.

The starch and sugar content were determined through Spectrophotometer by phenol–sulphuric acid reagent method as per the protocol depicted by Stickland and Wilson^30^ and Weng et al.^26^ with minor alterations.

### Statistical analysis

Analysis of variance (ANOVA) as suggested by Panse and Sukhatme (1985) was performed for statistical interpretation of data in Split Plot Design^32^. Standard error of mean (SEm ±) and critical difference (CD) at 5 percent level of significance were worked out for each character. The interaction analysis among different nutrient components and plant spacing was estimated using R programming software^26^.

### Ethical approval

This article does not contain any studies with human participants performed by any of the authors. The present investigation comprising plant studies was performed in accordance with relevant institutional, national, and international guidelines and legislation.

## Results

### Influence of spacing and deblossoming on nutritional value of tubers in tuber cowpea

The results presented in the Table 3 indicated that tuber cowpea planted at wider spacing S_4_ (60 cm × 30 cm) and S_3_ (60 cm × 15 cm) treatments, Table 3) accumulated significantly more total N (1.44, 1.31) respectively in the tubers as compared to tubers planted at closer spacings S_1_ (45 cm × 15 cm) and S_2_ (45 cm × 30 cm), Table 3). Likewise, the tubers of tuber cowpea planted at wider spacings (S_4_ (60 cm × 30 cm) and S_3_ (60 cm × 15 cm)) showed significantly higher total P content (0.207, 0.205) respectively than at closer spacings S_1_ (45 cm × 15 cm) and S_2_ (45 cm × 30 cm) (Table 3). The total K content was found to be independent of spacing treatments. The Ca and Mg contents also exhibited no significant variations at variable spacings (Table 3). The tubers harvested from the closest spacing S_1_ (45 cm × 15 cm), (Table 3) depicted significantly higher total Fe (116.28) content than wider spacing (Table 3). Total Mn, Cu and Zn contents in the tubers of tuber cowpea were independent of spacing effects (Table 3).

**Table 3:** Influence of spacing and deblossoming on nutritional value of tubers in tuber cowpea.

Treatment	Total N (%)	Total P (%)	Total K (%)	Total Ca (%)	Total Mg (ppm)	Total Fe (ppm)	Total Mn (ppm)	Total Zn (ppm)	Total Cu (ppm)
Main plot (Spacing)									
S_1_ (45 cm × 15 cm)	1.01	0.190	1.30	1451.66	2604.76	116.28	53.26	27.89	3.83
S_2_ (45 cm × 30 cm)	1.18	0.187	1.14	1186.71	2423.58	87.98	51.84	27.21	3.15
S_3_ (60 cm × 15 cm)	1.31	0.205	1.20	1060.94	2139.32	71.09	62.68	26.98	3.70
S_4_ (60 cm × 30 cm)	1.44	0.207	1.17	1352.94	2693.40	62.54	52.64	29.39	3.43
SE (m) ±	0.056	0.005	0.046	56.115	109.086	7.290	7.165	0.860	0.261
C.D. at 5%	0.196	0.016	NS	197.958	384.824	25.717	NS	NS	NS
Sub plot (Deblossoming)									
F_1_ (Deblossoming)	1.34	0.199	1.17	1282.29	2403.06	79.69	53.25	27.43	3.76
F_2_ (Blossom retention)	1.13	0.195	1.23	1243.84	2527.47	89.25	56.95	28.30	3.29
SE (m) ±	0.097	0.004	0.033	46.204	95.144	8.073	4.942	1.027	0.167
C.D. at 5%	NS	NS	NS	NS	NS	NS	NS	NS	NS
Interactions									
S_1_F_1_	1.13	0.197	1.23	1533.53	2591.83	101.47	38.78	26.21	4.37
S_1_F_2_	0.90	0.183	1.36	1369.79	2617.68	131.09	67.73	29.58	3.29
S_2_F_1_	1.32	0.190	1.17	1122.60	2325.55	83.83	54.73	28.17	3.28
S_2_F_2_	1.04	0.183	1.11	1250.83	2521.60	92.12	48.94	26.24	3.01
S_3_F_1_	1.30	0.200	1.17	1062.82	2009.83	72.94	64.30	27.11	3.56
S_3_F_2_	1.32	0.210	1.23	1059.07	2268.81	69.24	61.07	26.84	3.84
S_4_F_1_	1.62	0.210	1.12	1410.21	2685.01	60.53	55.20	28.23	3.84
S_4_F_2_	1.26	0.203	1.22	1295.67	2701.78	64.54	50.07	30.54	3.02
SE (m) ±	0.113	0.0065	0.0655	82.7444	163.744	11.928	10.071	1.452	0.360
C.D. at 5%	NS	NS	NS	NS	NS	NS	NS	NS	NS

### Analysis of quality parameters of tubers in tuber cowpea

The results pertaining to influence of treatments under study on quality parameters of tubers in tuber cowpea are presented in Table 4. The results indicated that the planting of tuber cowpea at wider spacing exhibited higher protein percentage in the tubers than closer spacing (Table 4). The deblossoming of tuber cowpea resulted in higher accumulation of proteins in the tubers (Table 4). The results indicated that starch and sugar content in tubers were not significantly influenced by variable spacings (Table 4). No significant variations were found with respect to interaction effects of treatments on different quality parameters of tubers in tuber cowpea (Table 4).

**Table 4:** Analysis of quality parameters of tubers in tuber cowpea.

Treatment	Protein (%)	Starch (%)	Sugar (%)
Main plot (Spacing)			
S_1_ (45 cm × 15 cm)	6.34	18.76	0.52
S_2_ (45 cm × 30 cm)	7.37	22.00	0.62
S_3_ (60 cm × 15 cm)	8.16	19.38	0.59
S_4_ (60 cm × 30 cm)	9.00	19.40	0.60
SE (m) ±	0.342	0.955	0.035
C.D. at 5%	1.207	NS	NS
Sub plot (Deblossoming)			
F_1_ (Deblossoming)	8.38	18.38	0.60
F_2_ (Blossom retention)	7.06	21.39	0.56
SE (m) ±	0.602	0.896	0.039
C.D. at 5%	NS	2.967	NS
Interactions			
S_1_F_1_	7.05	17.16	0.633
S_1_F_2_	5.63	20.35	0.410
S_2_F_1_	8.22	19.88	0.613
S_2_F_2_	6.52	24.12	0.620
S_3_F_1_	8.11	19.32	0.577
S_3_F_2_	8.21	19.45	0.597
S_4_F_1_	10.12	17.16	0.593
S_4_F_2_	7.88	21.64	0.607
SE (m) ±	0.701	1.468	0.058
C.D. at 5%	NS	NS	NS

### Influence of spacing and deblossoming on nutritional value of green pods in tuber cowpea

The results presented in Table 5 indicated that the total N and P contents in pods were found to be independent of spacing treatments. The spacings S_1_ (45 cm × 15 cm) and S_3_ (60 cm × 15 cm) depicted significantly higher total K content (0.61, 0.61) than spacings S_2_ (45 cmx 30 cm) and S_4_ (60 cm × 30 cm). Ca, Mg, Fe, Mn and Zn contents also exhibited no significant variations at variable spacings (Table 5). At spacing of S_3_ (60 cm × 15 cm), there was significantly higher Cu content (3.29) in the pods than at other spacings (Table 5). In general, the quality parameters were found to be independent of variable spacings in tuber cowpea. Similarly, the contents of majority of micronutrients under study were not affected by interactions of spacing and deblossoming treatments (Table 5).

**Table 5:** Influence of spacing and deblossoming on nutritional value of pod in tuber cowpea.

Treatment	Total N (%)	Total P (%)	Total K (%)	Total Ca (ppm)	Total Mg (ppm)	Total Fe (ppm)	Total Mn (ppm)	Total Zn (ppm)	Total Cu (ppm)
Main plot (Spacing)									
S_1_ (45 cm × 15 cm)	2.10	0.18	0.61	392.99	860.39	33.79	37.58	21.95	3.04
S_2_ (45 cm × 30 cm)	2.13	0.21	0.58	413.86	854.05	33.69	27.84	21.59	2.87
S_3_ (60 cm × 15 cm)	2.09	0.21	0.61	415.90	865.06	37.32	35.08	22.33	3.29
S_4_ (60 cm × 30 cm)	2.07	0.20	0.55	351.48	805.52	31.66	36.67	21.16	2.89
SE(m) ±	0.031	0.011	0.007	23.715	34.482	2.953	8.389	0.665	0.072
C.D. at 5%	NS	NS	0.023	NS	NS	NS	NS	NS	0.255
Sub plot (Deblossoming)									
F_1_ (Deblossoming)	0.00	0.00	0.00	0.00	0.00	0.00	0.00	0.00	0.00
F_2_ (Blossom retention)	4.19	0.40	1.17	787.12	1692.51	68.21	68.59	43.51	6.08
SE (m) ±	0.021	0.009	0.006	15.949	22.431	2.072	5.497	0.466	0.050
C.D. at 5%	0.070	0.028	0.020	52.820	74.285	6.861	18.205	1.542	0.166
Interactions									
S_1_F_1_	0.00	0.00	0.00	0.00	0.00	0.00	0.00	0.00	0.00
S_1_F_2_	4.19	0.36	1.21	785.99	1720.77	67.50	75.16	43.90	6.08
S_2_F_1_	0.00	0.00	0.00	0.00	0.00	0.00	0.00	0.00	0.00
S_2_F_2_	4.26	0.42	1.16	827.73	1708.09	67.38	55.68	43.18	5.74
S_3_F_1_	0.00	0.00	0.00	0.00	0.00	0.00	0.00	0.00	0.00
S_3_F_2_	4.18	0.43	1.21	831.80	1730.12	74.64	70.15	44.66	6.58
S_4_F_1_	0.00	0.00	0.00	0.00	0.00	0.00	0.00	0.00	0.00
S_4_F_2_	4.14	0.41	1.10	702.96	1611.04	63.32	73.35	42.31	5.77
SE (m) ±	0.043	0.016	0.011	33.134	47.8095	4.168	11.651	0.938	0.1015
C.D. at 5%	NS	NS	0.0395	NS	NS	NS	NS	NS	0.345

### Influence of spacing and deblossoming on protein content (%) of pods in tuber cowpea

The results pertaining to influence of treatments under study on protein content (%) of pod in tuber cowpea are presented in Table 6. The results indicated that protein contents in pods were not significantly influenced by variable spacings (Table 6). Greater accumulation of protein was found in F2 (blossom retention) (Table 6). Presence of blossoms may have been instrumental in greater accumulation of protein in the pods. The present findings are in agreement with those of Hussain and Basahy^33^, Gerrano et al.^34^, and Jayathilake et al.^35^.

**Table 6:** Influence of spacing and deblossoming on protein content (%) of pod in tuber cowpea.

Treatment	Protein (%) in pod
Main plot (Spacing)	
S_1_ (45 cm × 15 cm)	13.09
S_2_ (45 cm × 30 cm)	13.29
S_3_ (60 cm × 15 cm)	13.07
S_4_ (60 cm × 30 cm)	12.94
SE (m) ±	0.189
C.D. at 5%	NS
Sub plot (Deblossoming)	
F_1_ (Deblossoming)	0.00
F_2_ (Blossom retention)	26.19
SE (m) ±	0.131
C.D. at 5%	0.433
Interactions	
S_1_F_1_	0.00
S_1_F_2_	26.18
S_2_F_1_	0.00
S_2_F_2_	26.58
S_3_F_1_	0.00
S_3_F_2_	26.14
S_4_F_1_	0.00
S_4_F_2_	25.87
SE (m) ±	0.2665
C.D. at 5%	NS

### Influence of spacing and deblossoming on nutrient content of pericarp in tuber cowpea

The results pertaining to influence of treatments under study on nutritional parameters of pericarp in tuber cowpea are presented in Table 7. The results revealed that total N, P, K, Ca, Fe, Mn, Zn, Cu content in pericarp was found independent of spacing treatments in the study (Table 7). Greater accumulation of N, P, K, Ca, Mg Fe, Mn, Zn, Cu was found under blossom retention conditions (F_2_) (Table 7) The interaction effects of S_1_F_1_ in the pericarp indicated higher accumulation of Mg (Table 7).

**Table 7:** Influence of spacing and deblossoming on nutrient content of pericarp in tuber cowpea.

Treatment	Total N (%)	Total P (%)	Total K (%)	Total Ca (ppm)	Total Mg (ppm)	Total Fe (ppm)	Total Mn (ppm)	Total Zn (ppm)	Total Cu (ppm)
Main plot (Spacing)									
S_1_ (45 cm × 15 cm)	0.65	0.07	0.52	2551.57	2685.42	59.11	174.17	11.64	3.88
S_2_ (45 cm × 30 cm)	0.51	0.07	0.50	2618.22	2645.31	81.97	221.51	8.22	3.38
S_3_ (60 cm × 15 cm)	0.54	0.06	0.52	1478.52	2170.72	91.05	186.56	11.92	3.29
S_4_ (60 cm × 30 cm)	0.42	0.06	0.44	1720.37	2129.83	53.32	264.19	9.34	3.28
SE (m) ±	0.137	0.023	0.091	472.691	157.359	14.780	56.317	1.312	0.305
C.D. at 5%	NS	NS	NS	NS	NS	NS	NS	NS	NS
Sub plot (Deblossoming)									
F_1_ (Deblossoming)	0.00	0.00	0.00	0.00	0.00	0.00	0.00	0.00	0.00
F_2_ (Blossom retention)	1.06	0.13	0.99	4184.33	4815.64	142.72	423.21	20.56	6.91
SE (m) ±	0.107	0.015	0.060	305.861	103.886	10.363	36.016	0.900	0.228
C.D. at 5%	0.355	0.049	0.198	1012.940	344.047	34.321	119.277	2.980	0.756
Interactions									
S_1_F_1_	0.00	0.00	0.00	0.00	0.00	0.00	0.00	0.00	0.00
S_1_F_2_	1.29	0.13	1.04	5103.13	5370.85	118.21	348.34	23.28	7.77
S_2_F_1_	0.00	0.00	0.00	0.00	0.00	0.00	0.00	0.00	0.00
S_2_F_2_	1.03	0.14	1.00	5236.43	5290.63	163.94	443.02	16.44	6.77
S_3_F_1_	0.00	0.00	0.00	0.00	0.00	0.00	0.00	0.00	0.00
S_3_F_2_	1.08	0.11	1.05	2957.04	4341.44	182.10	373.11	23.84	6.58
S_4_F_1_	0.00	0.00	0.00	0.00	0.00	0.00	0.00	0.00	0.00
S_4_F_2_	0.84	0.12	0.89	3440.73	4259.65	106.64	528.38	18.68	6.53
SE (m) ±	0.1985	0.0315	0.1265	654.6095	218.911	20.8575	77.789	1.842	0.4375
C.D. at 5%	NS	NS	NS	NS	724.936	NS	NS	NS	NS

### Influence of spacing and deblossoming on protein (%) of pericarp in tuber cowpea

The results pertaining to influence of treatments under study on the Protein % of pericarp in the pods of tuber cowpea are presented in Table 8. The results depicted no significant effect on the protein content of pericarp under variable spacing. However, the higher value of total protein accumulation in the pericarp was observed in F_2_treatment (6.63%) (Table 8). While interaction treatments have also exhibited no significant effect on pericarp protein accumulation (Table 8).

**Table 8:** Influence of spacing and deblossoming on protein (%) of pericarp in tuber cowpea.

Treatment	Protein (%) in pericarp
Main plot (Spacing)	
S_1_ (45 cm × 15 cm)	4.04
S_2_ (45 cm × 30 cm)	3.22
S_3_ (60 cm × 15 cm)	3.37
S_4_ (60 cm × 30 cm)	2.63
SE (m) ±	0.857
C.D. at 5%	NS
Sub plot (Deblossoming)	
F_1_ (Deblossoming)	0.00
F_2_ (Blossom retention)	6.63
SE (m) ±	0.671
C.D. at 5%	2.222
Interactions	
S_1_F_1_	0.00
S_1_F_2_	8.08
S_2_F_1_	0.00
S_2_F_2_	6.43
S_3_F_1_	0.00
S_3_F_2_	6.74
S_4_F_1_	0.00
S_4_F_2_	5.25
SE (m) ±	1.2445
C.D. at 5%	NS

### Soil parameters affected by tuber cowpea

Initial soil analysis showed pH-5.63, EC-0.26, Organic carbon-0.61%, N-136.44 kg/ha, P-112.06 kg/ha, K-134.14 kg/ha, Fe-89.79 ppm, Cu-1.80 ppm, Zn-1.26 ppm and Mn-73.47 ppm. The results presented in Table 9 revealed that final soil parameters like pH, EC, Organic carbon, N, P, K, S, Fe, Cu, Mn, Zn, Ca, Mg exhibited no significant variations due to treatments. Irrespective of treatments, growing tuber cowpea raised the soil pH and increased the N, P, K, Fe and Cu contents of soil.

**Table 9:** Final soil parameters as affected by tuber cowpea at variable spacings and deblossoming treatments.

Treatment	pH	EC	Organic carbon (%)	N (kg/ha)	P (kg/ha)	K (kg/ha)	S (ppm)	Fe (ppm)	Cu (ppm)	Mn (ppm)	Zn (ppm)	Ca (ppm)	Mg (ppm)
Main plot (Spacing)													
S_1_ (45 cm × 15 cm)	5.80	0.19	0.58	222.74	160.46	206.27	7.83	100.57	5.50	79.62	3.04	19.43	0.73
S_2_ (45 cm×30 cm)	5.76	0.12	0.47	176.39	127.90	212.69	7.26	99.37	5.40	68.33	2.85	19.94	0.74
S_3_ (60 cm × 15 cm)	5.75	0.16	0.39	227.80	157.62	230.50	8.02	96.19	4.98	71.40	2.40	20.97	0.70
S_4_ (60 cm × 30 cm)	5.80	0.14	0.48	191.42	136.57	223.55	6.86	97.04	4.71	72.31	2.28	20.44	0.66
SE (m) ±	0.049	0.023	0.148	16.879	10.788	15.974	0.250	3.813	0.431	6.303	0.503	0.637	0.090
C.D. at 5%	NS	NS	NS	NS	NS	NS	NS	NS	NS	NS	NS	NS	NS
Sub plot (Deblossoming)													
F_1_ (Deblossoming)	5.76	0.18	0.47	202.36	137.55	231.24	7.78	98.44	4.97	75.69	2.59	20.31	0.68
F_2_ (Blossom retention)	5.79	0.12	0.48	206.82	153.72	205.26	7.20	98.15	5.33	70.14 +	2.69	20.08	0.74
SE (m) ±	0.044	0.013	0.110	10.369	10.542	11.821	0.364	1.546	0.208	3.799	0.158	0.235	0.028
C.D. at 5%	NS	0.044	NS	NS	NS	NS	NS	NS	NS	NS	NS	NS	NS
Interactions													
S_1_F_1_	5.77	0.25	0.36	225.21	167.48	238.86	8.65	97.75	4.83	87.24	3.27	19.88	0.74
S_1_F_2_	5.82	0.12	0.79	220.27	153.44	173.67	7.00	103.40	6.17	72.00	2.80	18.97	0.72
S_2_F_1_	5.68	0.13	0.58	185.69	100.95	223.62	8.35	101.17	5.41	68.40	2.76	20.35	0.68
S_2_F_2_	5.83	0.11	0.36	167.08	154.86	201.75	6.18	97.57	5.39	68.25	2.94	19.53	0.79
S_3_F_1_	5.81	0.17	0.41	223.49	151.27	242.29	7.67	100.03	5.13	75.35	2.27	20.68	0.68
S_3_F_2_	5.69	0.14	0.37	232.11	163.97	218.70	8.38	92.36	4.83	67.45	2.53	21.27	0.72
S_4_F_1_	5.78	0.15	0.55	175.03	130.52	220.19	6.47	94.83	4.50	71.79	2.07	20.33	0.72
S_4_F_2_	5.81	0.12	0.42	207.81	142.61	226.91	7.25	99.25	4.92	72.84	2.48	20.55	0.71
SE (m) ±	0.0745	0.0305	0.212	23.115	16.83	22.856	0.462	4.894	0.565	8.598	0.630	0.81	0.112
C.D. at 5%	NS	NS	NS	NS	NS	NS	NS	NS	NS	NS	NS	NS	NS

### Correlation analysis

**Figure 2:**
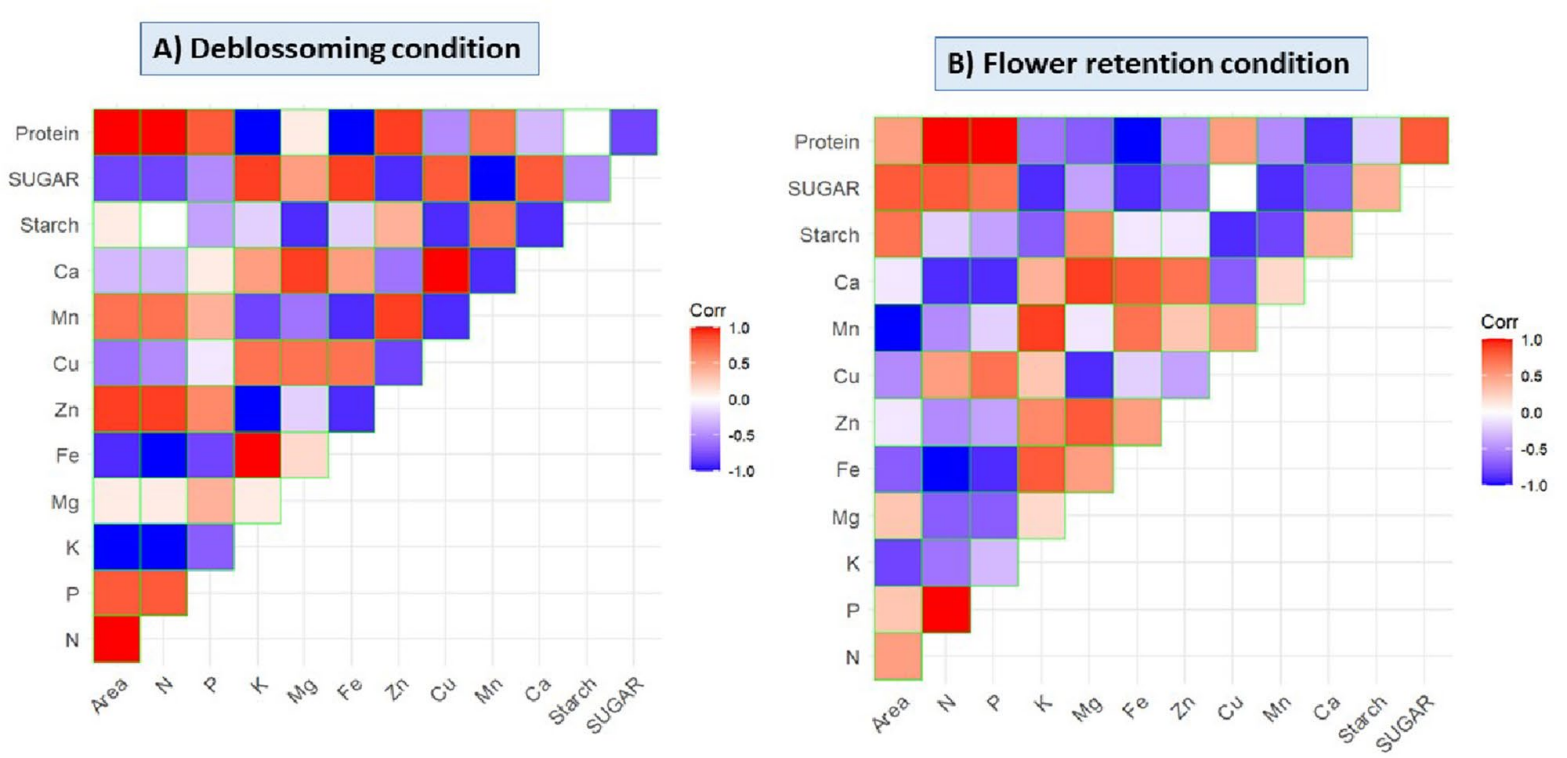
Corrplot (**A**) corrplot under flower deblossoming condition (**B**) corrplot under flower retention condition.

The results pertaining to correlation analysis among plant spacings and nutrient components have been presented in Tables 10, 11 and Fig. 2. The interaction analysis revealed the positive association between wide spacing and nitrogen (N), phosphorus (P), Manganese (Mn) and protein content of tubers under deblossoming condition. When flowers were not removed, the positive association was observed among area and N, P, Zn, Mn, Ca, Starch, Sugar and protein content. Under both conditions the plant spacing was negatively associated with potassium content.

**Table 10:**
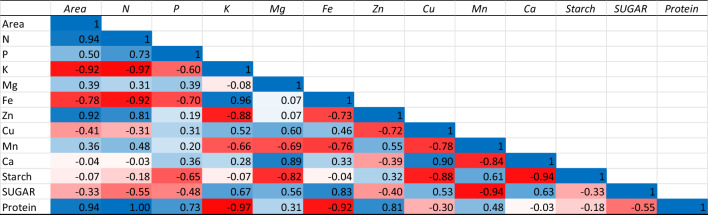
Interaction among nutrient components and plant spacing under deblossoming condition.

**Table 11:**
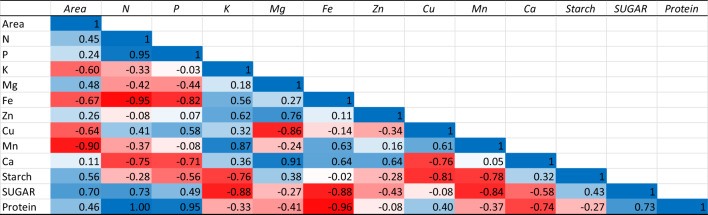
Interaction among nutrient components and plant spacing under flower retention condition.

## Discussion

The flowers are the essential plant organs influencing nutritional composition, rate of sugar and dry matter accumulation, fruit quality traits, and physiological processes in the plants^25^. In the tuber cowpea, the simultaneous formation of tubers and flowers occurs, and the substances accumulated are transported to these organs, flowers, and tubers, respectively. Hence, the nutritional status of pods, pericarp and tubers was compared in the present investigation under different conditions. The total N and P content of tubers increased with the increase in plant spacing of tuber cowpea. Maximum nitrogen concentration was observed at the high spacing of tuberose^36^. In rice, higher P uptake was reported by Parvin et al.^37^ at wider spacing^37^. Higher Zn and Mg contents of tubers were also observed at the widest spacing. At wider spacings, plants can absorb more water and nutrients, mainly because each plant produces a greater number of roots and has access to relatively greater proportions of soil and nutrients than a closely spaced plant. The highest zinc uptake in rice was also observed with the widest spacing, while the lowest uptake was recorded with the closest spacing^38^. Similar findings were reported by other researchers^39,40^. Magnesium is largely involved in chlorophyll synthesis, production, transportation, utilization of photo-assimilates, enzyme activation and protein synthesis^41^. Therefore, the higher protein content of tubers at wider spacing is also attributable to the higher content of Mg in tuber harvested from widely spaced plants.

In the present study, the wider row spacing of 60 cm was significantly better than 45 cm with respect to tuber protein content. The relatively higher levels of zinc observed in the tubers harvested from widely spaced plants, may also have been instrumental in increasing the protein content of such tubers, since zinc is known to be an essential component of thousands of proteins in plants^42^. Likewise, the higher accumulation of nutrients like N was reported recently in broccoli under wider spacing by Fitsum et al. and Abhijithnaik et al.^43,44^. The range of starch recorded in tuber cowpea is similar to that observed by other researchers^45–47^. The accumulation of more dry matter in the tuberous roots was probably accomplished by translocation of reserve carbohydrates from other plant parts.

Under the deblossoming conditions, the inhibitory effects of flower and pod development on vegetative growth and tuber yield of zombi pea might be due to direct competition for photosynthates linked food reserves between different plant organs^25^. The results obtained in the present investigation substantiate this hypothesis. In general, the flower initiation process removes a huge quantity of nitrogen and carbohydrates. The consumed nitrogen is exploited in the development of pods and seeds^27,48^. The interaction analysis revealed that plant spacing was positively correlated with N, P and protein content in the tubers of zombi pea under deblossoming conditions. A large quantity of elaborated nitrogen and carbohydrates are required in tuber development. Thus, under wider spacing with deblossoming conditions, the more food material elaborated to tuber formation and enlargement in zombi pea, and resulting in well-developed tubers and a high quantitative accumulation of protein content in the tubers. Further, it might be supported by the results of Roberts and Struckmeyer^49^ who recorded an increased phloem development in nonflowering plants as compared to flowering plants. In addition, the histological studies are required in zombi pea to analyze the alteration in blossoming and deblossoming conditions with respect to phloem development and flowering state. The mechanism involved when both tubers and flowers were produced in the same plant of Zombi pea can be explained by the fact that, there was unequal partitioning and translocation of assimilates involving those of carbon and of nitrogen. Larger proportions of carbon-based photo assimilate were translocated to the root tubers while greater amounts of nitrogen based assimilates were translocated to the fruits. It is possible that the inhibitory effects of floral and fruit development on vegetativeness and tuber development may be due to a simple direct competition for elaborated food materials between the vegetative organs, floral differentiation and fruit formation^24^. In legumes sucrose is the pre- dominant sugar and among nitrogenous solutes are the amino acids^50^. Sugars and other macromolecules arrive in heterotrophic plant tissues through the phloem^50^. Proteins are mobile in phloem and there may also be destination-selective translocation^31^. It was proposed that the difference in osmotic pressure between the phloem and the surrounding tissues could drive the unloading of solutes in sinks.

Forney and Breen^51^ also reported that the starch content of roots of deblossomed strawberry plants was about 18 times higher than plants with fruit^51^. The contents of P, Ca, Zn, Mg, Fe, Cu in green pod were maximum in the treatment combination S3F2 (wider spacing with inflorescence retention). It is attributed to the fact that wider spacings allow each plant to produce more roots and get access to greater quantity of nutrients per unit area of soil. The relatively higher levels of copper observed in different plant parts like tuber, pod and pericarp at the closest spacing (S1) may be explained by the greater concentrations of copper recorded in the soil at the same spacing. Copper acts as a cofactor in various enzymes and performs essential roles in photosynthesis, respiration and the electron transport chain, and is a structural component of defense genes. Excess of Cu, however, imparts negative effects on plant growth and productivity^52^. Irrespective of treatments, growing tuber cowpea raised the soil pH and increased the N, P, K, Fe and Cu contents of soil. Such enhancements may prove to be beneficial for the succeeding crops, with the exception of Fe and Cu which may prove to be toxic at higher concentrations.

## Conclusion

The present investigation clearly depicted the effect of plant spacing, blossom retention and deblossoming on nutritional status of tuber cowpea. In principle, the high accumulation of micronutrients viz. the total K, Mg, Fe, Mn, Zn and starch contents was observed in the tubers when flowers were retained on the plants. For developing the tubers with high protein content, the wider spacing (60 cm × 30 cm) with deblossoming can be recommended in zombi pea. In line with this the contents of P, Ca, Zn, Mg, Fe, Cu in green pods and pericarp were also maximum in the wider spacing with inflorescence retention.. Based on the results obtained in studying Zombi pea, we recommend that wider spacing (60 cm × 30 cm) can be adopted for higher protein content in tubers with deblossoming condition. The study revealed that different plant parts of zombi pea comprising pods, pericarp and tubers are enriched with nutrients. Hence, Zombi pea can be a potential commercial crop for combating the malnutrition problem and ill-effects of climate change in the pantropical regions like India and could be a wonder crop in enhancing food security as evident from its nutritional status.

### Supplementary Information

Supplementary Information.

## Data Availability

All data generated or analyzed during this study are included in this published article.
