# Supplementary material for: Nutritional status of Zombi pea (Vigna vexillata) as influenced by plant density and deblossoming
Source: Sci Rep. 2024 Feb 7;14:3189. doi: 10.1038/s41598-024-52736-7 (PMC10850079; doi:10.1038/s41598-024-52736-7)
Supplement: Supplementary file 1 — Supplementary Information. [file 41598_2024_52736_MOESM1_ESM.docx]

**Supplementary Table 1**. List of abbreviations used in the present publication

| % | Percentage |
| --- | --- |
| @ | At the rate |
| K | Potassium |
| Mg | Magnesium |
| Fe | Iron |
| Mn | Manganese |
| Zn | Zinc |
| N | Nitrogen |
| P | Phosphorus |
| Cu | Copper |
| Ca | Calcium |
| CD | Critical difference |
| SE (m) | Standard error of mean |
| Fig. | Figure |
| Ppm | Parts per million |
| NS | Non-significant |
| µg | Microgram |
| g | Gram |
| C | Carbon |
| ICRISAT | International Crops Research Institute for the Semi-Arid Tropics |
| cm | Centimeter |
| EC | Electrical conductivity |
| Kg | Kilogram |
| Ha | Hectare |
| S | Sulphur |
|  |  |
